# Substituting Fat with Olive Oil, Mash Potato, or a Gelatin Matrix in Low-Salt-Content Dry-Fermented Sausages

**DOI:** 10.3390/foods11182833

**Published:** 2022-09-13

**Authors:** Begoña Panea, Guillermo Ripoll

**Affiliations:** Centro de Investigación y Tecnología Agroalimentaria de Aragón (CITA), Instituto Agroalimentario de Aragón—IA2 (CITA-Universidad de Zaragoza), Avda. Montañana 930, 50059 Zaragoza, Spain

**Keywords:** fermented sausages, sensory acceptability, salt reduction, fat substitution, Spanish “chorizo”

## Abstract

It was investigated whether physicochemical properties and consumer perception of dry-fermented sausages were affected by the partial replacement of fat and salt by other compounds. A control batch and nine experimental batches, following 3 × 3 factorial design, were manufactured. The NaCl was replaced with of calcium lactate, magnesium chloride, or a blend 85% NaCl (sodium chloride) and 15% KCl (potassium chloride). The fat was partially substituted by olive oil, potato puree, or commercial gelatin. The pH, dry matter, fat content, free fatty acid content, peroxide index, microbial analysis, and tasting, were measured. Both fat type and slat type were affected to measured variables. In terms of moisture, gelatin would be the most recommendable substitute for fat, whereas KCL would be the better substitute for salt. The three oil batches and the potato-magnesium batches presented lower fat content than the control batch. Both the free fatty acid content and the peroxide values increased over the ripening time. At the end of the ripening, the three oil batches presented the highest values for free fatty acids, but there were no differences among the batches of peroxide index. Regarding bacterial counts, the potato-KCL batch was the most like the control batch. In visual appraisal, none of the scores of the measured variables were affected by the batch. Nevertheless, the batch of oil-magnesium would be purchased less than expected. The tasting quality was affected only by the salt type, but all of the batches were different from the control. Neither the gender (*p* > 0.05) nor the age (*p* > 0.05) of the respondents affected the taste scores, visual appraisal, or purchase intent. Results shows that the partial substitution of fat and salt in dry-fermented sausages is possible, even in greater percentages than those stated in the literature, without a detriment of sensory properties. Nevertheless, further experiments would be necessary to adjust the formulation, as well as to explore other possibilities.

## 1. Introduction

Fermented dry sausages, such as Spanish “chorizo”, are very popular and appreciated products in Spain and in other countries, mainly due to their sensory characteristics. The quality of meat products depends on their formulation, including salt and fat. Salt is an essential ingredient in cured raw meat products because it guarantees an adequate texture and flavor and controls the growth of pathogenic microorganisms [1]. Although excessive consumption of salt is detrimental to human health, a reduction of salt in dry sausages could be a major problem for the industry as it might cause a loss of quality (shelf-life, texture, flavor, and consumer acceptability) and create technological and food security problems [2]. One possibility is to partially replace NaCl with other compounds [3]. The most used substitutes are other chloride salts, such as KCl, CaCl_2_, or MgCl_2_, which show similar physicochemical properties to NaCl, but could decrease the salty taste and increase a bitter or metallic taste [4].

On their own, fats are a fundamental component of diet as they provide energy, are part of the cell membrane, and are the vehicle for fat-soluble vitamins and hormones. In food, in addition to their formulative properties, fats provide unctuosity and juiciness and contribute to overall texture. However, people generally perceive fats negatively and as being related to obesity, cancer, and cardiovascular diseases; therefore, a fat reduction is desirable from a health point of view. The reduction of saturated fat content can be achieved by substituting fat for oils [5,6], nuts [7,8], or carbohydrates, in place of animal fat [9].

Olive oil has been proposed as a good fat substitute due to its superior sensory and functional properties [10]. Nonetheless, some studies state that liquid oil has some technological problems when added directly as a substitute, and there is no consensus about the influence on sensory properties [11]. Hence, several techniques, such as emulsions [12,13,14,15] or imbibition into another matrix [16,17], have been suggested as a solution. Though they were usually employed in cooked or minced and/or cooked products, such as Frankfurters [6] or Bolognese-type sausage [18], when it was added to dry-fermented sausages oil olive was usually pre-emulsified with soy protein [19,20,21]. Notwithstanding, it has been demonstrated that the incorporation of soy modifies the quality characteristics of dry-fermented sausages and weakens its nutritional value [22]. Even when oils were pre-treated, the result depends on the applied treatment. For example, Josquin et al. [23] used encapsulated or emulsified fish oil, and whereas encapsulation resulted in a higher protein content and moisture percentage, the emulsion resulted in lower moisture percentages and no differences in protein content; therefore, the subject of how oils interact with the matrix is not completely resolve. In addition, it should be considered that as simple as some of these techniques may seem, they require the use of additives or special technical steps and, consequently, they are not within the reach of all companies, especially when speaking of small and artisan factories.

With regards to carbohydrates, several compounds has been proposed as fat substitute, such as Konjac gel [16,17,24], carrageenan gelled emulsion [15,25], fruit flavors [26], starch [25,27], soybean [14], chia [12,28], or celluloses [29,30]. The main advantage of carbohydrates was that oxidation is not compromised [15]. However, the main disadvantages consisted of nutritional weakness due to a decrease in protein content [16], as well as a hardening of the product [25], which could to lead to unfavorable sensory properties and causing rejection by consumers accustomed to particular organoleptic characteristics specific to each food [23,31].

Finally, most of studies concerning fat or salt substitution are focused on non-fermented products, such as Frankfurters or other type of cooked products, with the literature concerning dry-fermented sausages being limited. In addition, and to the best of our knowledge, most of the studies regarding dry-fermented sausages are centered on the replacement of salt or fat and only a few focuse on the concomitant replacement of both, fat and salt [11,22,32].

Therefore, the aim of this study was to investigate whether physicochemical properties and consumer perception of dry-fermented sausages (Spanish “chorizo”) were affected by the partial replacement of fat and salt by other compounds.

## 2. Materials and Methods

### 2.1. Sausage Processing and Sampling

All species and additives used in the preparation of the different sausages were purchased in a local supermarket (Alcampo, Valencia, Spain).

Dry sausages were manufactured in the facilities of Embutidos Manolica (Andorra, Teruel, Spain). A control batch and nine experimental batches were manufactured.

All 10 batches weighed 26.5 kg/each. The control batch was manufactured using lean pork-leg (37.7%), flank (56.6%), NaCl (1.5%), water (1.5%), paprika (2.2%), potassium nitrate (0.3%), and a 0.1% commercial antioxidant-stabilizing blend including E451, E452, E331, and E301 (Productos Pilarica, S.A., Paterna, Spain). The nine experimental batches follow a 3 × 3 factorial design. The NaCl was replaced with the same amount (1.5%) of calcium lactate (Merck, Darmstadt, Germany, CAS no. 5743-47-5), magnesium chloride (Merck, Darmstadt, Germany, CAS no. 7786-30-3), or a blend 85% NaCl:15% KCl (Jesús Navarro, S.A., Novelda, Spain). The flank proportion was reduced to 47.2%, with the remaining 9.4% comprised of olive oil (La Española, Acesur, Sevilla, Spain), potato puree (Maggi, Nestlé, Vevey, Suiza), or gelatin (Royal, Mondelez Int., Chicago, IL, USA). The potato puree and the gelatin were prepared following the manufacturer’s instructions before being added to the mixture. Briefly, for the puree, mix 500 mL of water and 250 mL of semi-skimmed milk, heat to boiling, remove from the heat and dissolve the potato flakes in the liquid, allow to stand for 2 min and stir the puree with the help of a spoon. For the gelatin, dissolve the contents of the gelatin sachet in ¾ liter of boiling water, stir until completely dissolved, add ¾ liter of cold boiled water and stir. The other ingredients (water, paprika, potassium nitrate, and antioxidant blend) were added in the same proportion described in the control batch.

In all batches, the meat was minced and trimmed with a mincer (TECMAQ Alimat 130/2, Barcelona, Spain) fitted with a grille with 8 mm diameter vents, and then mixed with the other ingredients and kneaded for 3 min with a blender machine (FUERPLA, Valencia, Spain). All batches were stored at 2 °C for 24 h, and then stuffed (REX RVF 327REX Technologie GmbH and Co KG, Thalgau, Austria) into 32–34 mm diameter natural pig casings (Vicente Gallent, Valencia, Spain) to form pieces of 170 g. A sample of five sausages per batch was collected at this point to measure the initial values for dry matter, free fatty acid content, and peroxide value.

**Figure 1:**
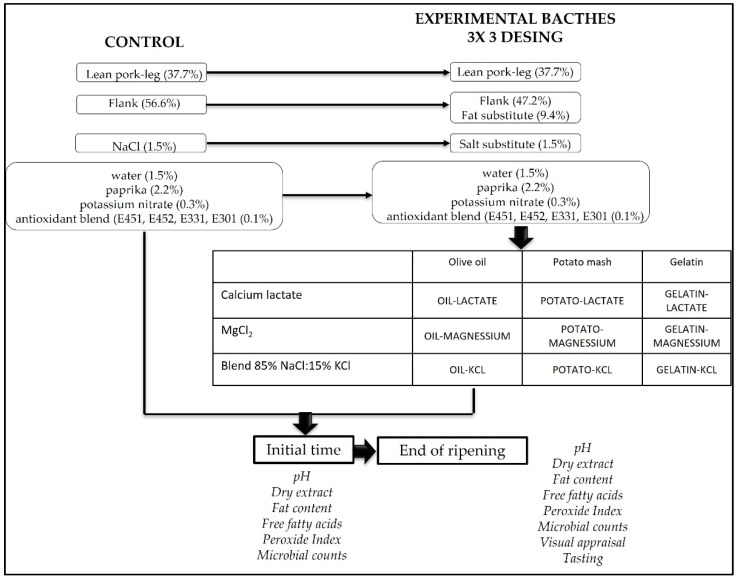
Experimental design.

Then, the sausages were hung to dry for 28 days, maintained at 75% relative humidity at 3 °C, without heat or forced air, which is a typical management. Weekly, a sausage from each batch was weighed and checked for optimal drying. Once the sausages reached the specific final weight loss (30%), they were sampled. All sausages in each batch were cut into four portions, vacuum packed (MCOEX material, Coimbra Pack, S.L., Zaragoza, Spain), and randomly assigned to the different analyses. The following analyses were performed: pH, dry matter, fat content, free fatty acid content, peroxide index, microbial analysis, and tasting. The experimental design is shown in Figure 1.

### 2.2. Methodological Procedures

The pH was measured in a homogenate prepared by blending 20 g of sausage with 80 mL of distilled water for 30 s [33]. Measurements were made in triplicate with a pH meter equipped with a Crison 507 penetrating electrode (Crison Instruments S.A., Barcelona, Spain). To calculate the dry matter, the samples were dried in an oven (Memmert UFP 600, Schwabach, Germany) at 105 °C until constant weight and calculated as a percentage of the initial sample weight [8]. Fat content was determined following Spanish Official Methods [34]. Lipids were extracted with chloroform samples to determine the free fatty acid concentration and the peroxide value was determined following the methodology described by Aguirrezábal et al. [35]. In brief, the determination of total free fatty acids was performed by titration of 10 mL of the lipid extract using phenolphthalein as an indicator, and the free fatty acid content was expressed as g of oleic acid/100 g of sausage, whereas for the peroxide value, the lipid extract was titrated with 0.002 M Na_2_S_2_O_7_, using starch as an indicator, and the results are expressed as meq of oxygen/kg of sausage.

### 2.3. Microbial Analysis

Samples for total aerobic mesophilic and lactic acid bacteria (LAB) were taken on the 1st and 30th days of ripening. For microbiological analysis, an internal procedure based on ISO 15214:1998 [36] was used. A 10 g sample of sausage was aseptically weighed in a sterile plastic bag. Subsequently, samples were homogenized with 90 mL of a sterile solution of 0.1% (*w*/*v*) peptone water (Oxoid, Unipath, Basingtoke, UK) for 2 min at 20–25 °C in a Masticator blender (IUL Instruments, Barcelona, Spain), thus making a 1/10 dilution. Serial 10-fold dilutions were prepared by mixing 1 mL of the previous dilution with 9 mL of 0.1% (*w*/*v*) sterile peptone water. Total aerobic mesophilic bacteria were enumerated following standard ISO 4833, 2003 (colony-count technique at 30 °C), by aseptically spread plating 1 mL of each of the serial 10-fold dilutions on Plate Count Agar (PCA). After incubation at 30 °C for 72 h, the CFU per gram of sample was determined. For lactic acid bacteria enumeration, 100 mL samples of similar 10-fold dilutions were plated on Man, Rogosa, and Sharpe (MRS) agar. After incubation at 35 °C for 3 days or 30 °C for 5 days in an aerobic atmosphere supplemented with 5% carbon dioxide, the cfu per gram of sample was determined.

### 2.4. Consumer Appraisal

Two consumer tests were conducted: a visual appreciation with real product; and a home-test sensory assessment. For the two tests, students and workers who had no connection to this research were recruited from the Aula Dei Campus (Zaragoza, Spain). Personal data such as identification or electronic mail were not needed, and there was no financial compensation. Participants were clearly informed of the aim of the study and gave implicit consent for research use of the supplied information, according to European regulations and ESOMAR guidelines [37].

For visual appraisal, samples were chopped into 2 cm-thick pieces. Then, samples were placed on a polystyrene tray (three pieces per tray) overwrapped with a polyethylene low density (PE-LD) oxygen permeable film (Coimbra Pack, S.L., Zaragoza, Spain) without contact with the surface. Trays were identified with two-digit random numbers and randomly placed in a Carrier Multinor 1540/80 refrigerated island display case (Carrier Refrigeración Ibérica SA, Madrid, Spain) with a display area of 1 m2 (1.3 m × 0.8 m) at 0–2 °C. Samples were available from 08:00 to 16:00 and, to avoid effects related to the order of presentation and first-order and carry-over effects, they were moved randomly three times during the test day. Lighting was provided by LED bulbs with a luminous flux of 816 lumen, a color temperature of 4000 K, a color rendering index >80 and a standard deviation color matching equal to 3 MacAdam ellipses [38]. The illuminance on the surface of the samples was approximately 1300 lx, ensuring the minimum level of illuminance in areas with high visual requirements. Each consumer was provided with a form on which they were asked about their gender and age. Then, they were asked to evaluate from 1 (very bad) to 10 (exceptionally good) the general appearance of the samples. Additionally, they were asked about their purchase intention (yes/no). Consumers were not given any information about the samples. A total of 47 consumers (38% men) completed the test. By age, 6% were ≤25 years old, 21% were 26–40 years old, 53% were 41–55 years old, and the other 19% were >55 years old.

A home-test was performed to investigate consumer appraisal. A balance design of incomplete blocks was used. Each consumer received a sachet with two different samples, previously identified by a two-figure random number, and their accompanying evaluation questionnaire. Consumers were not given any information about the samples. Consumers were asked about sausage color, smell, taste, and texture (hardness) using a 10-point scale (from 1 = dislike very much to 10 = like very much). Additionally, consumers were invited to include, in a blank, unsolicited adjectives or commentaries about the sausages. Finally, consumer gender and age were recorded. A total of 161 respondents (55% men) completed the test. By age, 13% were ≤25 years old, 27% were 26–40 years old, 32% were 41–55 years old, and the other 28% were >55 years old.

### 2.5. Statistical Analysis

Statistical analysis was performed using the XLSTAT 2020 software (Addinsoft, Inc., Brooklyn, NY, USA). An ANOVA procedure was conducted to investigate the effect of ripening on dry matter, free fatty acid content, peroxide value, and microbial counts. A two-way ANOVA procedure was conducted to investigate differences between batches for all the studied variables. The least squares mean (LSM) was separated using Tukey’s *t*-test. All statistical tests of LSM were performed for a significance level *p* < 0.05.

## 3. Results

### 3.1. Physicochemical Analysis: pH, Dry Matter, Fat Content, Free Fatty Acid Content, and Peroxide Index

The end of drying was established at 30% depletion. The average weight loss was 31.8%, and there were no differences between batches (*p* = 0.814). The average final pH was 5.41 (s.e. = 0.047) without differences between batches (*p* > 0.05). The pH values agreed with those reported by other authors for dry-fermented sausages [39,40,41]. Lack of differences in the pH when salt or fat was replaced agreed with the conclusions of other authors [9,40,42].

At the beginning of the experiment (Table 1), the average dry matter was 50.5% (s.e. = 0.51), and at the end of curing the average dry matter value was 79.6% (s.e. = 0.37). Current results for dry matter are in accordance with those described by Mora-Gallego et al. [43] and by Gomez and Lorenzo [40], but are slightly higher than the 60% described by other authors [44,45]. Fat type affected the dry matter of both fresh and cured sausages (*p* < 0.05), whereas salt affected the dry matter only at the end of the ripening period (*p* < 0.05). In fresh sausages, the three oil batches presented higher dry matter than the potato or gelatin batches, and the values for the oil batches were like those of the control batch. Nevertheless, at the end of ripening, dry matter values tended to be lower in the oil batches than in the potato and gelatin batches. Regarding the salt effect at the end of the ripening process, independent of the fat type, KCL batches presented higher values than batches manufactured with MgCl_2_ (from now, magnesium) or batches manufactured with calcium lactate (from now, lactate). The result is not surprising, since this mixture still contains 85% NaCl; however, depending on the substitute for the fat, the mixture acts in one way or another and is or is not different from the control batch. So, as can be seen, no differences were found between the control batch and the potato-KCL batch, or between the control batch and the gelatin-KCL batch, but differences exist between the control batch and the oil-KCL batch.

The mean values for fat content are shown in Table 2. The control showed a higher fat content (56.2%) than the others, but it was only significantly different from the three oil batches and from the potato-magnesium batch. Moon et al. [46] stated that fat content increased from 1st to 15th days when a combination of oil and soy is added, which would be contrary to current results. Nevertheless, these mentioned authors concluded that differences are due to moisture loss and, as can be seen, the three oil batches and the potato-magnesium batches are those which presented the lowest final dry matter percentages. In addition, the interactions that occurred inside the matrix cannot be underestimated, since the matrix can encapsulate the fat. For example, in an experiment carried out on low-salt dry fermented sausages [22], in which the fat was partially replaced by soy, an interaction between the salt type and the fat source was found in the fat content, and the authors explained that soy protein acts as a fat encapsulating agent, forming a layer around the fat drops and protecting them, promoting protein-lipid interactions. Similarly, [24] working with sausage in which fish-oil was encapsulated on a konjac matrix, found that the higher the content oil, the lower the fat and moisture content. These findings imply that for further studies, the origin, nature, and scope of these interactions between the ingredients of the matrix should be studied.

Spanish regulation [33] stated that the final composition of this kind of sausage should have less than 57% fat, less than 2% reducing sugars, more than 30% protein, a collagen/protein relationship lower than 16%, and less than 1% added proteins. All our sausages fulfilled these requirements. The results obtained for fat content were higher than those described by other authors, ranging from 12% to 35% [1,43,47,48,49], but agree with the values of approximately 50% shown by Gómez et al. [39]. Both the fat type and salt affected the fat content of the sausages, as expected [27,40].

The results for free fatty acids and the peroxide index are shown in Table 3. Lipid oxidation in dry-fermented sausages is influenced by a wide range of factors, including the composition of the raw material and the characteristics of additives. Regarding composition, since pork contains a significantly high proportion of phospholipid, they are especially prone to oxidation [50].

Free fatty acids are generated by the lipolysis phenomena that occurs during the ripening process [40], and they are important because they are linked to lipid oxidation, which can contribute to the flavor. At the initial time, the free fatty acid content was lower for the control and gelatin-magnesium batches, than for the other batches, and the oil batches tended to present the highest values. At the end, the three oil batches clearly presented the highest values, in agreement with Gomez and Lorenzo [40], who reported that sausages with 10% fat presented lower values of free fatty acids than samples with 20% or 30% fat. As expected, free fatty acids increased over the ripening time [39].

Peroxide gives the initial evidence of rancidity and is the most widely used method to measure oxidation at the first stages of storage. With storage time, the peroxides changed into other compounds, and for the final stages other methods, such as T-bar, are recommended. No differences between the batches were found at the start for peroxide values, indicating a lack of fat degradation, as expected. The peroxide index values increased over time, as expected [39,45]. At the end of ripening, the oil-lactate, oil-KCL and gelatin-lactate batches presented values higher than the potato-lactate batch. It has been stated that the direct addition of olive oil resulted in higher T-bar values [51] but in the current experiment the differences between batches were slight, and none of them presented values significantly different from the control batch. The current results for the peroxide index values agreed with those reported by Panea and Ripoll [22] in sausages, in which fat was partially substituted by soy (from 0.17 to 3.27 meqO_2_/kg) or by Lekjing [52] in cooked pork sausages (from 1.6 to 2.8 meqO_2_/kg). Regarding salt, some studies indicated that NaCl could promote the oxidation of meat products due to a disruption of cell integrity, and favoring contact of the oxidizer with the substrates, as well as being due to a release of iron, which acts as a catalyzer [53,54], whereas the use of KCl reduces the oxidation rate [50]. However, the current results showed no differences between NaCl and the other salt types. In this way, a peroxide level of 4.0 meqO_2_/kg has been identified as the upper limit for acceptable quality in dry sausages [55], and all of our formulae were below that threshold.

### 3.2. Microbiology

Total mesophilic aerobic and lactic acid bacteria (hereafter BAL) were counted (Table 4). Total mesophilic are indicators of hygiene conditions during the process. Lactic acid bacteria are developed throughout the curing process; they reduce the pH of sausage by producing lactic acid, which helps its preservation, as many bacteria are sensitive to low pH.

The mean values at the beginning were 5.4 log UFC/g for total mesophilic and 4.9 log UFC/g for LAB. At the final time, the global means for microbial counts were 8.2 log UFC/g for total mesophilic and 8.1 log UFC/g for LAB. The current values for microbial counts agreed with those presented by other authors in similar products [45,56,57].

As expected, all the microbial counts increased with ripening time. Nevertheless, at the initial time, no differences between batches were found for any of the two microbial groups (*p* < 0.05), indicating that all batches started from the same situation and allowed us to clearly discriminate the effect of the fat type and salt substitution.

At the final time, differences were found between batches for the two bacterial groups (*p* < 0.05). Regarding total mesophilic, the three oil batches, as well as the gelatin-lactate batch, presented the highest values, whereas the potato-KCL presented the lowest values, although no differences were found between potato-KCL and the control batches. Concerning LAB, only the three potato batches were not significantly different from the control batch, as the values for these mentioned batches were lower than the values of the other batches.

Triki, et al. [9] found that sausage formulation did not affect microbial counts. Similarly, Lizaso et al. [45] reported that LAB counts were approximately 10^7^ UFC/g, without changes over the ripening time. Nevertheless, Liaros et al. [58] reported that LAB counts increased from 5.5 log UFC/g to 8.5 log UFC/g in the first 4 days and remained constant thereafter, and [59] reported an increase in LAB counts from 6.1 log UFC/g to 8.3 lof UFC/g.

Gimeno et al. [42] reported that salt substitution (KCl or CaCl_2_) resulted in higher LAB counts, although other authors [59] have not found differences in function due to the salt type used. In the current experiment, the effect of salt substitution on LAB counts depended on the fat substitute; LAB counts were higher than those of the control batch in the three oil batches and in the three gelatin batches, but not in the three potato batches.

### 3.3. Consumer Tests

#### 3.3.1. Visual

The results of the visual test are shown in Table 5. Fat type affected both scores and purchase intention (*p* < 0.001), whereas salt type did not (*p* > 0.05). The oil-magnesium batch tended to present the lowest scores, whereas the gelatin-potassium batch tended to present the highest scores, and in general, the three oil batches scored lower than the others. Nevertheless, none of the batches were statistically different from the control batch. On the other hand, when respondents were asked about their purchase intention, only half (48.9%) indicated that they would buy sausages, with differences among batches (*p* = 0.006). Therefore, the oil-magnesium batch would be chosen to be less appreciated than expected.

Neither the gender (*p* > 0.05) nor the age (*p* > 0.05) of the respondents affected either the scores or the purchase intention.

It has been reported that low-fat sausages presented low L* values and high a* values due to their lower moisture content [15,24], although Bloukas et al. [51] reported lighter color in sausages formulated with olive oil than in control sausages. Yet, it has been stated that for consumers, the rugose surface and not the changes in color were the determinants for the rejection of the visual appraisal of these sausages [58]. This could explain why some authors, such as Backes et al. [60], concluded that the partial replacement of the fat by canola-oil unaffected visual scores.

#### 3.3.2. Tasting

The means for scores in a taste test are shown in Table 6. Fat type did not affect any of measured variables (*p* > 0.05), whereas salt type affected the flavor (*p* < 0.05), but differences were found only for oil batches, with the oil-magnesium batch presenting lower values than the oil-potassium batch. Nevertheless, none of the nine experimental batches were different, for any of the considered variables, from the control batch.

None of the scores of the measured variables were affected by the gender or age of the respondents (*p* > 0.05).

The survey included a blank space to fill with unsolicited adjectives. Forty-two percent of the comments were texture defects, 8% were curing defects, 6% were taste defects, 2% were odor defects, and 1% were color defects.

The effect of fat substitution on sensory characteristics has been reported by several authors [40]. Triki et al. [9] reported that the use of a konjac gel to partially replace the pork backfat resulted in a decrease in the hardness measured in a texture profile analysis, whereas other authors [47,58] reported that when fat was partially replaced, sausages became tougher than the control group, and taste perception was worse. Similarly, Campagnol et al. [59] reported that when fat was replaced by polysaccharides, the flavor intensity decreased. Nevertheless, we have found no effect on the fat substitution, which could be because the effect of fat substitution depends enormously on the salt type used, as we observed in previous experiments [22,32].

Regarding the salt type, it has been reported that KCl has a bitter taste, and consequently, a limit of 50% substitution has been proposed [61]. In this way, some studies [4] stated that replacing 50% NaCl with KCl decreased the sensory quality. Nevertheless, Dos Santos [62] reported that replacement with KCl had no effect on volatile compounds. In our experiment, the use of KCl did not affect the sensory quality of the sausage, maybe because the KCl was a commercial blend 85% NaCl:15% KCL, and, consequently, the actual percentage of substitution was lower than the limit of 50%.

Similarly, the use of MgCl_2_ is limited because it gives a bitter-salt taste [63] and even metallic and astringent sensations [64], whereas it was reported that the addition of CaCl_2_ is acceptable up to 5% [65] and it improved the generation of hexanal, heptanal, and other volatile compounds [62]. Choi et al. [66] used a blend of 30% K-lactate and 10% calcium ascorbate in Frankfurt sausages and found no differences in either hardness or flavor, although the chewiness decreased, and the juiciness and saltiness increased in sausages that were substituted. In contrast, other authors reported that substitution with K-lactate did not affect sensory attributes [67]. Then, it seems that the behavior of the salt substitution depends not only on the percentage of substitution, but in the way in which the substitute was added, as well as the specific geometry of the matrix.

## 4. Conclusions

From the results of this study, we concluded that both the fat type and the salt type affected to measured variables. In terms of moisture, gelatin would be the most recommendable substitute for fat, whereas KCL would be the better substitute for salt. The three oil batches and the potato-magnesium batches presented lower fat content that the control batch. Both free fatty acid content and the peroxide values increased over the ripening time. At the end of the ripening, the three oil batches presented the highest values for free fatty acids, but there were no differences among batches of the peroxide index. Regarding bacterial counts, the potato-KCL batch was the most like the control batch. None of the scores of the measured variables in visual appraisal were affected by the batch. Nevertheless, the oil-magnesium batch would be purchased less than expected. Tasting quality was affected only by salt type, but all of the batches were different from the control. Neither the gender (*p* > 0.05) nor the age (*p* > 0.05) of the respondents affected the taste scores, visual appraisal, or purchase intent. The results show that partial substitution of fat and salt in dry-fermented sausages is possible, even in greater percentages than stated in the literature, without a detriment to sensory properties. Nevertheless, further experiments would be necessary to adjust the formulation, as well as to explore other possibilities.

## Figures and Tables

**Table 1:** The means and standard error for the dry matter (%) of 10 experimental batches of dry-fermented sausages and the *p* values for studied effects (fat type and salt type).

Batch		Initial Dry Matter (%)	Final Dry Matter (%)
CONTROL		53.4 ^a^	81.7 ^ab^
OIL	LACTATE	52.6 ^a^	74.4 ^f^
	MAGNESIUM	53.9 ^a^	75.9 ^ef^
	KCL	52.1 ^abc^	78.9 ^cd^
POTATO	LACTATE	49.0 ^cde^	80.1 ^bc^
	MAGNESIUM	50.1 ^bcd^	77.5 ^de^
	KCL	49.5 ^bcd^	83.2 ^b^
GELATIN	LACTATE	48.7 ^de^	79.9 ^bc^
	MAGNESIUM	49.5 ^bcd^	80.6 ^bc^
	KCL	46.0 ^e^	83.9 ^a^
Standard error		0.9	0.7
Fat type effect (*p* value)		<0.001	<0.001
Salt type effect (*p* value)		0.076	<0.001
Fat type x salt type (*p* value)		0.546	0.083

Lactate-potassium lactate; Magnesium-magnesium chloride; KCL-potassium chloride. Different letters indicate significant differences between batches (*p* < 0.05).

**Table 2:** The means and standard error for fat amount of 10 experimental batches of dry-fermented sausages and the *p* values for studied effects (fat type and salt type).

Batch		Fat Content (%)
CONTROL		56.2 ^a^
OIL	LACTATE	49.5 ^c^
	MAGNESIUM	48.8 ^c^
	KCL	51.9 ^c^
POTATO	LACTATE	54.4 ^ab^
	MAGNESIUM	53.6 ^bc^
	KCL	55.8 ^a^
GELATIN	LACTATE	55.7 ^a^
	MAGNESIUM	54.3 ^ab^
	KCL	53.8 ^b^
Standard error		0.599
Fat type effect (*p* value)		<0.001
Salt type effect (*p* value)		<0.001
Fat type x salt type (*p* value)		0.006

Lactate-potassium lactate; Magnesium-magnesium chloride; KCL-potassium chloride. Different letters indicate significant differences between batches (*p* < 0.05).

**Table 3:** The means and standard error for free fatty acid content and for peroxide value of 10 experimental batches of dry-fermented sausages and the *p* values for studied effects (fat type and salt type).

		Free Fatty Acids (g Oleic Acid/100 g)		Peroxide Index (meqO_2_/kg)	
Batch		Initial	Final	Initial	Final
CONTROL		0.11 ^c^	0.57 ^d^	1.28	3.04 ^ab^
OIL	LACTATE	0.23 ^a^	2.80 ^a^	2.16	3.88 ^a^
	MAGNESIUM	0.16 ^ab^	2.34 ^c^	1.25	2.59 ^ab^
	KCL	0.25 ^a^	2.58 ^b^	1.71	3.78 ^a^
POTATO	LACTATE	0.18 ^b^	0.66 ^d^	1.94	2.02 ^b^
	MAGNESIUM	0.14 ^ab^	0.55 ^d^	1.93	2.50 ^ab^
	KCL	0.15 ^ab^	0.61 ^d^	2.44	3.32 ^ab^
GELATIN	LACTATE	0.14 ^ab^	0.67 ^d^	1.98	3.77 ^a^
	MAGNESIUM	0.12 ^c^	0.58 ^d^	1.43	3.06 ^ab^
	KCL	0.13 ^ab^	0.67 ^d^	1.96	3.10 ^ab^
Standard error		0.085	0.129	0.115	0.146
Fat type effect		<0.001	<0.001	0.494	0.043
Salt type effect (*p* value)		0.045	<0.001	0.404	0.129
Fat type x salt type (*p* value)		0.237	0.006	0.772	0.093

Lactate-potassium lactate; Magnesium-magnesium chloride; KCL-potassium chloride. Different letters indicate significant differences between batches (*p* < 0.05).

**Table 4:** The means and standard error for microbial counts of 10 experimental batches of dry-fermented sausages and the *p* values for studied effects (fat type and salt type).

		Total Mesophilic (log UFC/g)		LAB (log UFC/g)	
Batch		Initial	Final	Initial	Final
CONTROL		5.5	7.9 ^ef^	4.5	7.9 ^c^
OIL	LACTATE	5.4	8.5 ^a^	5.0	8.5 ^a^
	MAGNESIUM	5.5	8.6 ^a^	5.0	8.6 ^a^
	KCL	5.5	8.4 ^ab^	5.1	8.5 ^ab^
POTATO	LACTATE	5.4	8.0 ^de^	4.8	7.9 ^c^
	MAGNESIUM	5.5	8.0 ^de^	5.0	7.9 ^c^
	KCL	5.5	7.7 ^f^	4.9	7.7 ^c^
GELATIN	LACTATE	5.5	8.5 ^a^	5.1	8.5 ^ab^
	MAGNESIUM	5.6	8.3 ^bc^	5.1	8.2 ^b^
	KCL	5.1	8.2 ^cd^	4.8	8.2 ^b^
Standard error		0.034	0.032	0.054	0.036
Fat type effect		0.252	<0.001	0.547	<0.001
Salt type effect (*p* value)		0.156	<0.001	0.827	0.014
Fat type x salt type (*p* value)		0.094	0.005	0.690	0.019

Lactate-potassium lactate; Magnesium-magnesium chloride; KCL-potassium chloride. Different letters indicate significant differences between batches (*p* < 0.05).

**Table 5:** The means and standard error for scores in a visual appraisal test and the percentages of purchase intention based on the visual appraisal for 10 experimental batches of dry-fermented sausages. *p* values for batch effect and for consumer gender and age effects.

		Visual Appraisal Test	
Batch		Score (1–10)	Purchase Intention (% yes)
CONTROL		6.0 ^abc^	55.0
OIL	LACTATE	5.6 ^abc^	43.5
	MAGNESIUM	4.4 ^c^	10.0
	KCL	4.7 ^bc^	30.0
POTATO	LACTATE	6.6 ^a^	52.9
	MAGNESIUM	6.8 ^a^	68.4
	KCL	6.3 ^ab^	64.7
GELATIN	LACTATE	6.3 ^abc^	63.2
	MAGNESIUM	6.3 ^ab^	47.1
	KCL	6.7 ^a^	62.5
Standard error		0.134	-
Fat type effect		<0.001	<0.001
Salt type effect (*p* value)		0.512	0.557
Fat type x salt type (*p* value)		0.283	0.274
Consumer’ gender effect (*p* value)		0.840	0.406
Consumer’ age effect (*p* value)		0.271	0.207

Lactate-potassium lactate; Magnesium-magnesium chloride; KCL-potassium chloride. Different letters indicate significant differences between batches (*p* < 0.05).

**Table 6:** The means and standard error for scores (1–10) in a taste test of 10 experimental batches of dry-fermented sausages. *p*-values for salt type and fat type effects and for consumer’ gender and age effects.

	Taste Test			
Batch	Color	Flavor	Taste	Texture
CONTROL	6.3	6.0 ^ab^	6.6	6.0
OIL-LACTATE	6.2	6.2 ^ab^	5.6	5.3
OIL-MAGNESIUM	5.9	5.7 ^b^	5.4	5.0
OIL-KCL	6.0	6.7 ^a^	5.8	5.1
POTATO-LACTATE	6.3	5.6 ^b^	5.8	5.4
POTATO-MAGNESIUM	5.9	5.5 ^b^	5.8	5.0
POTATO-KCL	6.1	5.6 ^b^	5.3	5.2
GELATIN-LACTATE	6.0	5.8 ^ab^	5.7	5.6
GELATIN-MAGNESIUM	5.8	5.4 ^b^	5.2	5.4
GELATIN-KCL	6.5	6.3 ab	6.0	6.2
Standard error	0.092	0.101	0.104	0.108
Fat type effect	0.981	0.067	0.997	0.059
Salt type effect (*p* value)	0.300	0.027	0.578	0.147
Fat type x salt type (*p* value)	0.698	0.590	0.354	0.679
Consumer’ gender effect (*p* value)	0.103	0.106	0.927	0.510
Consumer’ age effect (*p* value)	0.486	0.109	0.980	0.204

Lactate-potassium lactate; Magnesium-magnesium chloride; KCL-potassium chloride; means in bold are different from control. Effects in bold are significant (*p* < 0.05); Different letters mean statistical differences between batches (*p* < 0.05).

## Data Availability

The data used to support the findings of this study can be made available by the corresponding author upon request.
